# Medical Items and News of Interest to the Physician

**Published:** 1872-01

**Authors:** 


### Medical Items and News,

### Of Interest to the Profession.

CULLED FROM THE STANDARD MEDICAL JOURNALS OF THE WORLD.

NOTE.—These formulæ are intended only for the regular practitioner of medicine, and should be employed only by him, or under his immediate supervision.

**Small Pox—**

In Philadelphia has caused the death of 293 persons up to Nov. 1st. 1,647 cases were then reported.

**Yellow Fever—**

Still continues at this date (Nov. 20th,) at Charleston, S. C., and Key West. Vessels from this port are quarentined in New York harbor.

**To Test Alcohol.**

Add to the alcohol a piece of caustic potash. If any foreign matter be present, the alcohol will become more or less yellow; if it remains colorless, it is pure.

**Medical Director.**

William M. Wood has resigned, and Medical Director Jonathan M. Foltz has been appointed in his place as Chief of the Bureau of Medicine and Surgery in Washington. D. C.

**Whooping Cough.**

Dr. Miller, in the *American Practitioner*, finds bromide of ammonium, in combination with tincture of veratrum, exceedingly useful in whooping cough.

**Chronic Dysentery.**

Dr. C. Handfield Jones details a case (*British Med. Jour.*), in which the *ashantee* bark seemed of great service in a case of obstinate chronic dysentery, in the dose of half a fluid drachm of tincture three times a day.

**Chorea.**

Trousseau highly extols the use of strychnia in chorea of children. His favorite formula is as follows:

℞.
Strychniæ sulphat., gr. j.,
Syrp. simple., f. ℥ iiss. M.

A child ten years of age is given a teaspoonful two or three times a day, the dose being cautiously increased until it produces its physiological effects.—*Med. Record.*

**Scabies.**

Rub one drachm of the solution of carbolic acid with four ounces of lard. By the diligent rubbing of the affected part with this three times a day, the itch is usually cured in three or four days.—*Med. & Surg. Reporter.*

**Traumatic Erysipelas.**

Dr. Bonfingli, an Italian surgeon of note, recommends the use of the oil of turpentine in the treatment of traumatic erysipelas. He has met great success with this agent. He uses it as a dressing, applied by means of lint saturated with it and frequently changed.

**Dahlberg's Tincture.**

Tincture colocynthidis, known also as Dahlberg's tincture, is made as follows:

℞. Colocynth pulp., (cut small and free from seeds,) ℥ j; aniseed, ʒ j; proof spirit 1 ℔. Digest for eight days, express and filter. Dose, 6 to 20 drops.—*Pharm. Journal.*

**Chorea.**

Dr. John Rose reports the case of an anæmic girl, aged thirteen years, who had chorea following rheumatism, who was successfully treated by ether spray applied along the spine for four or five minutes each time, and after fifteen sittings, a very marked improvement took place, followed by complete recovery.—*Medical News.*

**Neuralgia.**

A case of obstinate neuralgia is related, which was cured by sulphate of nickel in doses of half a grain three times a day. At the end of one week, the dose was increased to one grain. Its sedative action was speedily manifested in reducing the pulse and procuring sleep; all symptoms of the paroxysm disappeared.—*Oregon Med. & Surg. Repository.*

**Dermato Syphilis.**

Prof. L. A. During, of the University of Penn., prescribed at a recent clinic, as reported in the *Med. Times*, as follows:

℞.
Potassii iodidi, ʒ v;
Tr. cinchonæ, comp.,
Syrp. simplicis, āā f. ℥ ij. M.

SIG.—A teaspoonful three times a day after meals. Crusts are to be removed from the ulcers by poultices, after which they are to be dressed with ungent. hydrarg.

**Fissure of Anus.**

M. Van Holsbeck states that he has succeeded in curing anal fissures with the following application: dissolve one part of tannic acid in sixteen parts of glycerine. A tent wet with this preparation is to be introduced into the rectum night and morning. The bowels are to be kept open.—*Medical News.*

**Tympanic Distention.**

At the meeting of the French Academy of Medicine for July, a communication was received from Prof. Fonnssagraves, of Montpellier, in which he strongly recommended puncture of the stomach and intestines whenever serious symptoms arise from distentiôn by gas. He asserted that the operation was perfectly safe, and could readily be accomplished with an ordinary, hydrocele, trocar and canula.

**Hemorrhage and Ipecac.**

Dr. Martin, of Indiana, (*N. Y. Med. Journal,*) reports a case of injury to the tonsil, attended with alarming hemorrhage, which was controlled by the administration of *small doses* of ipecac, until gentle vomiting ensued. Natural plugging followed after the contraction of tissues.—He has also tried it many times in obstinate contraction of the uterus, and never experienced ill effects.

**Pitting in Small Pox.**

In the Edinburgh *Medical Review*, Dr. W. Scott states he has been very successful in preventing disfigurement by small-pox, by keeping the face, from the first appearance of the eruption, constantly moist with a solution of carbolic acid in oil, (2 to 8). The application is very grateful to the patient, allaying the itching and irritation, and preventing the desire to scratch off the scabs.

**Whooping Cough.**

Dr. O. C. Farquhar, of Putnam, Ohio, writes to the Phila. *Med. & Surg. Reporter*, that he has derived great benefit from the use of sunflower seeds, in the treatment of whooping cough.

He takes one pound of the seeds, bruises them in a mortar, places them in a bottle with three pints of whisky, allowing them to digest for seven days. Of this, he places four ounces in a half pint of sweetened water, and to a child one year old, gives a tablespoonful every three hours, or oftener, as the urgency of the cough requires.

**Hæmorrhoids.**

Apply the following ointment morning and evening:

℞

Morphiæ sulph., gr. iij;

Ext. stramonii, gr. xxx:

Olei olivæ, gr. lx;

Plumbi oxy-carb., gr. lx;

Cerat. adipis, ʒ iij. M.

Fiat unguent.—*Richmond Med. Journal.*

**Gangrene.**

Dr. Lange mentions a case of gangrene in which turpentine, after other remedies had proved ineffectual, not only saved the patient's life, but also nearly all the parts affected. A girl eleven years old had gangrenous destruction of the right cheek. The part was treated with turpentine on lint, and the dressing changed every two hours.—*Indiana Jour. of Med.*

**Enlarged Tonsils.**

Dr. Rumbold, of St. Louis, has treated successfully a number of cases of enlarged tonsils by injecting the glands by means of a hypodermic syringe, with a solution of iodine—iodine, gr. ij; potass. iod., ℈ ij; aqua, ℥ j. Generally, a slight inflammation followed the injection, but soon subsided. From twelve to seventeen injections, ordinarily two a week, were sufficient to reduce the gland to its normal condition.—*Med. Press & Journal.*

**Bronchocele.**

Prof. James R. Wood, of New York, extols the following formula as an ointment in bronchocele and other glandular tumors:

℞

Ung. stramonii, ℥ ij;

Ext. conii, ʒ ij;

Iod. potassii, ʒ ij;

Iodidi, grs. x. M.

*Detroit Review of Med. & Pharm.*

**Obstinate Constipation.**

The *Bulletin General de Therapeutique*, for June 15th, contains the history of a case which had resisted all the usual remedies, and was finally cured by an occasional application of induced electricity. The negative pole of the battery was placed within the rectum and the positive over the umbilicus. The current was feeble at first and gradually increased. After twenty minutes application, a free evacuation was produced, giving the patient instant relief.

**Diphtheria.**

℞.

Carbolic acid, (sol.) ♏ xx;

Acetic acid, ʒ ss.;

Honey;

Tincture of Myrrh, āā f. ʒ ij.;

Water ad f. ℥. vj.

Let the acids be well shaken together before the other ingredients are added. Use as a gargle or apply to the throat with a probang.—*New Remedies.*

---

**Cod Liver Oil and Iodide of Iron.**

Dr. J. Cummiskey writes to the *Phila. Medical Times*, that he has been able to combine iodide of iron with ol. morrhuæ, in the following manner:—

℞.

Iodide of iron, gr. lxiv.;
Ether sulphuric, q. s.;
Cod-liver oil, (clarified,) Oj.

Dissolve in a mortar, the iodide of iron in a slight excess of ether, and add the oil gradually, stirring with the pestle rapidly until the mixture is complete. Keep in a tightly corked bottle. Every dr. of the oil contains ½ grain of the iodide of iron, which is the usual dose.

---

**Smallpox.**

Mr. Aikman, in the hemorrhagic form of smallpox, has observed the most beneficial results from the administration of two tablespoonfuls of the following mixture every three hours:

℞

Liquoris strychniæ;

Tinct. ferri muriat, āā ʒ j;

Inf. quassiæ, ad ℥ viij. M.

The action of the strychniæ, as a nerve tonic, is that which is chiefly sought. Mr. Aikman, as well as others of the medical attendants on smallpox in London, are much impressed with its value.—*Phila. Med. & Surg. Reporter.*

---

**Wet Sheets in Diarrhœa.**

Oppenheimer employed this treatment in twenty cases of rapid diarrhœa in children, from fourteen days to four years old, with three deaths and seventeen recoveries. In two cases of chronic diarrhœa there was no result. He used sheets wet in water at 50° to 55° Far., in which the little sufferer was enveloped and then covered with a blanket, which was kept on until perspiration set in—say from half an hour to an hour. Internally the patients were given ice water, broth, barley-water, milk, wine or brandy, but no medicine. As soon as perspiration occurred, the packing was removed, the child wiped dry, and cloths dipped in cold water laid upon its abdomen, and renewed as fast as they grew warm. When the diarrhœa persisted, the packing was repeated within an hour. If cerebral congestion occurred, the packing was discontinued and ice applied to the head. The treatment seemed suited only to acute attacks of diarrhœa in children.—*Schmidt's Jharbücher.*

---

**Wine of Beef and Iron.**

This formula is the same as used by the Newark Pharmaceutical Association:

℞.

Extracti carnis, (Liebigs,) 1 oz.;

Ferri citrat., 96 grs.;

Vini xerici, 16 oz.;

Syrupi, 2 oz.;

Pimentæ, (contus,) ½ dr.;

Aquæ, q. s. ft., 24 oz.

Dissolve the extract of beef in 4 oz. of water and add the allspice; after standing ten hours, add the wine and syrup, then the citrate of iron, previously dissolved in 2 oz. of water. Filter.

Each fluid ounce contains: Fresh beef 1 oz.; cirate of iron 4 gr. Dose—one tablespoonful.

---

**Frozen Beef Essence.**

Dr. Horace B. Hare, of Philadelphia, communicates an important discovery to the *Phila. Med. Times.* He says that often children who are suffering from scarlet fever, have great difficulty in swallowing food in consequence of sore throat. He has observed that ice is always taken eagerly by them. This suggested the idea of freezing beef essence and feeding it in such cases. The beef essence is made in the usual manner and frozen in an ice-cream freezer until solid. It is then fed to the patient in suitable quantities. The Doctor says it is always eagerly and easily swallowed, when all other forms of food are refused.

---

**Tubercular Phthisis.**

Dr. Van den Court, Professor of Clinical Medicine at the hospital of St. Jean, at Brussels, in a paper of some length commends most highly the following formula:

Pure Codliver Oil, 100 drachms.

Rub it with hydrated (slaked) lime, add little by little until it acquires the consistency of a pilular mass. Add to this,

Essential Oil of Bitter Almonds, 1 drachm.

Mix thoroughly and divide into boluses weighing from 20 to 25 centigrammes. These may be coated over with tolu by means of the etherial tincture, and placed in a powder composed of three parts of sugar and one part orris root. Two to be taken after each meal. Six to eight daily.—*Bull. Gen. de Therap.*

**Typhoid Fever.**

John E. Owen, M. D., in *Chicago Med. Journal*, observes that during the last four years, both in hospital and private practice, milk and the acid and strychnia mixture have been administered to patients with typhoid fever with success. The mixture is prepared as follows:

℞.

Acid sulp. arom. ʒ iij;

Strychniæ sulph. gr. ⅓;

Syrp. simpl., ℥ v. M.

DOSE.—A tablespoonful. There is one noticeable feature in cases treated with strychnia, viz., the dry brown tongue soon becomes moist, and remains so during the treatment; this is effected, he believes, mainly through the agency of strychnia, by increasing the nutrative and assimilative functions of the system.

**To Dissolve Santonin.**

Santonin is difficult of solution, but it may be accomplished in the following manner:

℞
Santonini, in pulvere, gr. xij;
Sodæ bicarbonatis, gr. xx;
Aquæ destillatæ, f. ℥ iij.

Put the soda and water into a flask, keep the fluid near the boiling point, adding as it disappears about two grains of the santonin at a time, until the whole is dissolved. Solution is effected in about half an hour, during which time the water is reduced to f. ℥ ij. If need be, reduce by boiling to this bulk, when f. ℥ j. will contain a full dose—six grains of santonin. If an alkaline reaction be objectionable, neutralize with acetic acid.—*Phila. Med. & Surg. Reporter.*

**Local Anæsthesia.**

Dr. Spessa states in the *Bulletin des Sc. Med.* (Italy), that he has succeeded in preventing pain, during the slitting of a fistulous tract, by injecting a solution of morphine into the tract before the use of the knife. The same author had occasion to touch the vulva vegetation of a young girl, with butter of antimony; the pain was very acute, but disappeared on the part being brushed over with a solution of morphine. A boy of fifteen, suffering from hip joint disease, required an issue over and behind the great trochanter. An injection of morphine was first made over the region, and Vienna paste applied, which latter remained about eight minutes. The paste did not give any pain.—*Medical Cosmos.*

**Peritonitis.**

Dr. Whitelaw commends the treatment of acute peritonitis by absolute quiet on the back in a cool, well ventilated room, 60° F., with a diet restricted to a little milk and lime water, thin arrow root, and tablespoonful doses of cold water, external applications, and the exhibition of teaspoonful doses of the following mixture every fifth hour, if pain be present.

℞.

Tinct. belladonnæ;

Sol. mur. morphiæ, āā ʒ ij;

Aquæ, ad. ℥ ij.

Sometimes for this he substitutes laudanum. His external applications are made in the following way:—A pailful of hot water is placed by the bed, and out of it a triple ply of flannel is wrung, laid on the abdomen, and entirely covered with a piece of silk-oil cloth, a four legged stool or hoop, or other contrivance, keeping off the bed clothes. As soon as the application becomes too cool to be agreeable to the patient, a fresh one is applied.—*Glasgow Med. Journal.*

**Cholera Infantum.**

Dr. J. E. Reeves recommends the following in the early stage of this affection:

℞

Plumb. acet., gr. xij;

Morph. acet., gr. ss;

Sacch. alb., ʒ j ss;

Acet. destill., ʒ ss;

Aquæ., f. ℥ ij. M.

Sig:—A teaspoonful to a child from one to two years old. Should this fail to check vomiting, ten to fifteen drops of "*chloroform paregoric*" may be given. After vomiting has ceased:

℞
Bismuth subnitr., gr. xxxvj;
Cretæ preparat., gr. xx;
Pulv. doveri, gr. v. M.

Sig—One every three or four hours until convalescence.—*Phila. Med. Times.*

**Calculus Nephritis.**

Dr. Wm. M. Chambers, of Effingham, Ill., (*Chicago Med. Examiner*,) reports a case in which recovery occurred from the following treatment:

Externally, a blister; internally, the following mixture:

℞.

Pot. acetatis, ℥ ss;
Vini colchici, f. ℥ ss;
Spts. æt, nit., f. ℥ ij;
Tinct. opii camph., f. ℥ ss;
Fld. ext. belladonnæ, ʒ j;
Aquæ cinnamoni, f. ℥ ss. M.

Sig:—One teaspoonful every three hours..

A calculus of phosphate of lime weighing five grains, passed the urethra the day following.

---

**Carbolic Acid as a Local Anæsthetic.**

Carbolic acid possesses wonderful powers as a local anæsthetic. If a portion of skin is covered with a cloth soaked in a solution of carbolic acid, for half an hour, and then a streak traced across the surface with a camel's hair brush dipped in acid liquified by one-twentieth its bulk of water, the skin may be divided along the course of the streak with a scalpel, quite down to the subcutaneous cellular tissue, without causing any pain. Abscesses, whitloes and buboes may be opened with great advantage in this manner. Sometimes it is necessary, when making an incision through the integument, to brush out the wound made with some liquified acid before making the incision deeper.—*Brathwaite's Retrospect.*

[In illustration of the wonderful anæsthetic powers of carbolic acid, we recently witnessed a case in this city, where a physician had applied the acid to the finger of a lady to allay the pain accompanying a severe whitloe. One drachm of the crystals of carbolic acid, were dissolved in eight ounces of water, and the patient directed to saturate a cloth with the solution and wrap the finger in it. The pain soon subsided, and in twenty-four hours no sensibility whatever was experienced in the finger, which so alarmed the patient that she called another physician, who, being ignorant of the action of carbolic acid, and finding some extravisated blood beneath the skin, causing slight discoloration of the parts, proclaimed the finger to be gangrenous, and absolutely recommended amputation. The attending physician, however, brought counsel to satisfy the patient's fears, and, by the aid of warm poultices, the finger was soon restored to sensibility, and in a few days the whitloe disappeared.—ED. BISTOURY.]

